# Updated Clinical Guidelines on the Management of Hepatitis C Infection in Children

**DOI:** 10.3390/pathogens13020180

**Published:** 2024-02-16

**Authors:** Chaowapong Jarasvaraparn, Christopher Hartley, Wikrom Karnsakul

**Affiliations:** 1 Division of Pediatric Gastroenterology, Hepatology and Nutrition, Indiana University School of Medicine, Indianapolis, IN 46201, USA; 2 Department of Pharmacy, The Johns Hopkins Hospital, Baltimore, MD 21287, USA; chartle8@jh.edu; 3 Division of Pediatric Gastroenterology, Hepatology and Nutrition, Department of Pediatrics, Johns Hopkins University School of Medicine, Baltimore, MD 21287, USA; wkarnsa1@jhmi.edu

**Keywords:** hepatitis C virus (HCV), NAT, nucleic acid test, DAAs, direct-acting antivirals, SVR12, sustained virological response at 12 weeks after treatment

## Abstract

Children represent only a small proportion of those infected with the hepatitis C virus (HCV) compared to adults. Nevertheless, a substantial number of children have chronic HCV infection and are at risk of complications including cirrhosis, portal hypertension, hepatic decompensation with hepatic encephalopathy, and hepatocellular carcinoma in adulthood. The overall prevalence of the HCV in children was estimated to be 0.87% worldwide. The HCV spreads through the blood. Children born to women with chronic hepatitis C should be evaluated and tested for HCV due to the known risk of infection. The course of treatment for hepatitis C depends on the type of HCV. Currently, there are two pan-genotype HCV treatments (Glecaprevir/pibrentasvir and Sofosbuvir/velpatasvir) for children. We aim to review the updated clinical guidelines on the management of HCV infection in children, including screening, diagnosis, and long-term monitoring, as well as currently published clinical trials and ongoing research on direct acting antiviral hepatitis C treatment in children.

## 1. Introduction

Alter H, Houghton M, and Rice C discovered the hepatitis C virus (HCV) and received the 2020 Nobel prize for their work. Alter H showed that non-A and non-B hepatitis were caused by a transmissible agent, Houghton M identified the HCV, and Rice C characterized the viral lifecycle and showed that the HCV caused liver injury. The HCV is a significant public health burden affecting 58 million people worldwide [1]. The percentage of people who are seropositive for anti-HCV antibodies worldwide is estimated to have increased from 2.3% to 2.8% between 1990 to 2005 [1]. Most patients (80–85%) cannot clear the virus themselves and progress to chronic infection including cirrhosis, portal hypertension, hepatic decompensation with hepatic encephalopathy, and hepatocellular carcinoma. Fortunately, children represent only a small proportion of HCV infections. Nevertheless, there was one report in 2014 showing that 11 million people with HCV infection worldwide were younger than 15 years of age [2]. In the United States, antibodies to the HCV are found in approximately 0.3% of children under 14 years old and 9% aged 15–24 years old, according to the CDC database in 2016.

The overall prevalence of the HCV in children was estimated to be 0.87%, ranging from 0.34% in Europe to 3.02% in Africa [3]. HCV prevalence was significantly higher in children older than 10 years (0.97%) when compared to those under 10 years old (0.75%, *p* < 0.001) [3]. The global estimate for viremic prevalence in the pediatric population aged 0–18 years was 0.13% (95% uncertainty interval 0.08–0.16), corresponding to 3.26 million (2.07–3.90) children with the HCV in 2018 [4]. The prevalence of children infected with the HCV is mostly reported in Asia, the Middle East, and Africa [4]. However, the incidence has been increasing in the United States due to illicit drug use. During 2021, 43 states reported a total of 107,300 newly identified chronic hepatitis C cases, corresponding to 39.8 chronic hepatitis C cases per 100,000 people from a recent CDC report [5]. The rate of acute HCV infection among young people and the prevalence of young adults using illicit substances are increasing in the United States, especially in suburban areas [6,7]. This could reflect the epidemic of prescription opioid and subsequent heroin use in young people in suburban areas. Based on a comprehensive literature review from 2000 to 2019 to determine historical HCV prevalence estimates in children in all 249 countries and territories of the world, HCV prevalence increased with age in all regions. With that said, the prevalence of HCV in adults was significantly associated with HCV prevalence in children aged 5–19 years (*p* < 0.0001), and the proportion of HCV infections in people who inject drugs was significantly associated with HCV prevalence in children aged 15–19 years (*p* = 0.036). Moreover, HCV prevalence in women of childbearing age was the strongest predictor of HCV prevalence in children aged 0–4 years (*p* < 0.0001). [4]. Since many children with HCV infection are infected from women of childbearing age by vertical transmission, pediatric cases are also expected to increase in the future.

Although HCV RNA can be detected in blood (both serum and plasma), saliva, tears, seminal fluid, ascitic fluid, and cerebrospinal fluid, perinatal transmission is the most common mode of HCV transmission in children, representing more than 1500 new cases per year in the United States, though many cases are undiagnosed [8]. The incidence of HCV transmission from a mother to a child is approximately 5% in HCV RNA-positive mothers; however, the risk is increased for mothers with untreated human immunodeficiency virus (HIV) coinfection, and higher HCV RNA levels (>6 log^10^/mL) [9,10]. The treatment of HIV/HCV coinfection in women reduces the rate of vertical transmission, and direct-acting antiviral agents (DAAs) for the HCV have reduced the rates of HCV in children when mothers with the HCV were treated prior to conception [10].

## 2. Natural History

The HCV is the most common blood-borne pathogen which leads to morbidity and mortality. HCV infection generally progresses slowly, so the consequences, including cirrhosis or hepatocellular carcinoma, are rare during childhood. HCV infection during infancy is most likely to clear spontaneously, with rates ranging from 20 to 45% [11,12,13,14] during the first two years of life. In contrast, HCV acquired later in life is less likely to clear spontaneously. Infants with perinatal HCV infection may have elevated serum aminotransferases for a few years and then normalize. Children who remain HCV RNA positive during or after the first year of life are less likely to clear the infection spontaneously. Interestingly, children presenting with symptomatic acute HCV infection may have a lower risk of developing chronic HCV infection [15]. Children with chronic HCV infection are usually asymptomatic; however, acute HCV infection may rarely cause malaise, nausea, right upper abdominal pain, jaundice, and dark urine.

The HCV is detected in plasma within days of exposure, often in 7–28 days. Viremia peaks in the first 8–12 weeks of infection and then drops to undetectable levels (viral clearance) in cases of spontaneous clearance. Chronic HCV infection appears to be due to weak CD4+ and CD8+ T-cell responses, which fail to control viral replication and lead to local inflammation that triggers fibrogenesis. The HCV may drive hepatocarcinogenesis directly via transmitting signals, and modulates hepatocyte gene expression following engagement with cellular receptors and/or indirectly through the induction of chronic liver inflammation [1]. The progression of advanced liver disease is not common in children and is more likely to occur in adults over 30 years old. However, some studies show progression to advanced liver disease and cirrhosis during childhood (Table 1). Currently, it is still unclear what factors are associated with the disease progression of HCV infection in children. We therefore cannot predict who will have a worse outcome during adulthood and who should be promptly treated to avoid complications such as cirrhosis, portal hypertension, hepatocellular carcinoma, and death. In the most updated classification, there are seven genotypes of the HCV based on their nucleotide variability in HCV sequences recovered from multiple geographic regions [2]:

- Genotype 1: Most common worldwide, 60–70% in the United States, more severe liver disease.
- Genotype 2: Most common in central and west Africa.
- Genotype 3: Widely distributed but most common in Asia, related to illicit drug use.
- Genotype 4: Northern Africa and the Middle East.
- Genotype 5: South Africa.
- Genotype 6: Southeast Asia.
- Genotype 7: Central Africa.

## 3. Screening

Proper identification of perinatally infected children, referral to care, and curative treatment are critical to achieving the goal of hepatitis C elimination. In 2020, the CDC released universal screening recommendations for adults due to continued increases in HCV infections [20], which included recommendations for screening for pregnant persons during each pregnancy [21]. The CDC also introduced four new recommendations:

(1) HCV testing of all perinatally exposed infants with a nucleic acid test (NAT) for detection of HCV RNA at 2–6 months of age.
(2) Consultation with a health care provider with expertise in pediatric hepatitis C management for all infants and children with detectable HCV RNA.
(3) Perinatally exposed infants and children with an undetectable HCV RNA result at or after 2 months of age do not require further follow up unless clinically warranted.
(4) A NAT for HCV RNA is recommended for perinatally exposed infants and children aged 7–17 months who previously have not been tested, and an anti-HCV antibody test followed by a reflex NAT for HCV RNA (when anti-HCV is reactive) is recommended for perinatally exposed children aged ≥18 months who previously have not been tested.

Selected screening in infants/children is appropriate for some groups who are at high risk of HCV infection, such as those with evidence of hepatitis, a history of mother’s HCV infection, international adoptees or refugees, children with HIV infection, children who are victims of a sexual assault, adolescents with a history of multiple sexual partners, and adolescents with a history of illicit drug use.

## 4. Diagnosis

The diagnosis of HCV infection is based on the detection of antibodies to recombinant HCV polypeptides and assays for HCV RNA. For children aged ≥ 18 months old, the initial evaluation is anti-HCV antibody testing. A reactive or indeterminate antibody test should be checked by HCV RNA testing. If HCV RNA is detected, indicating viremia/active disease, these patients should be further evaluated to determine the genotype and monitor for disease progression or spontaneous clearance. If the anti-HCV antibody test is positive and HCV RNA is not detected, this means either the patient has cleared the virus spontaneously or there was a false-positive antibody test.

**Figure 1:**
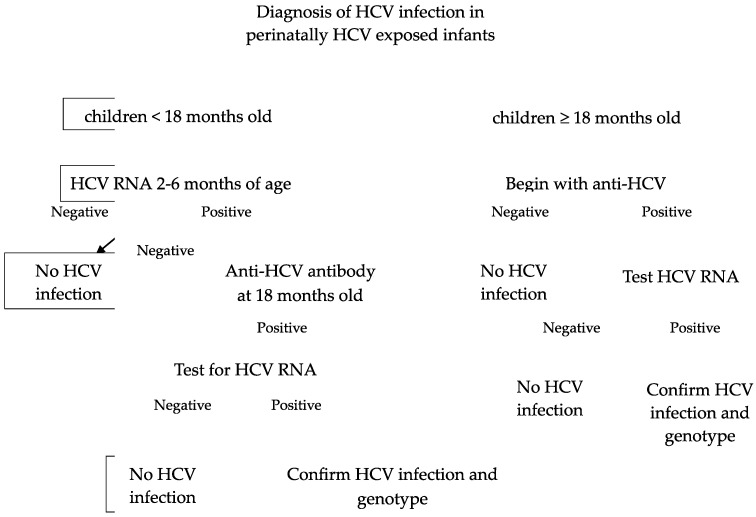
Diagnosis of HCV infection in perinatally HCV-exposed infants [24].

For children aged < 18 months old, the first step is testing for HCV RNA, ideally at 2 and 6 months of age. If it is not detected, this can reassure the family that HCV infection is very unlikely [22]. If HCV RNA is detected, it suggests that the infant has an infection, but it does not predict chronic infection because infants can clear HCV spontaneously. Repeating HCV RNA testing prior to 18 months of age is unnecessary. The next step is to check anti-HCV antibodies at 18 months of age for all perinatally exposed infants, and those with positive anti-HCV antibodies should be tested for HCV RNA. Prior to 18 months, anti-HCV antibodies are not specific because a positive test could reflect passive transfer of the maternal immunoglobulin G antibody (IgG Ab). Passively acquired maternal HCV antibodies are cleared in 95% of children by 12 months of age [23]. The diagram of HCV diagnosis in children is in Figure 1.

## 5. Further Evaluation

Further evaluation consists of checking the viral genotype. Currently, some of the direct-acting antivirals (DAAs) have activity against all known HCV genotypes; however, many public and private treatment programs still require HCV genotyping prior to approving treatment. Some DAAs are pangenotypic (Mavyret^®^ AbbVie Inc., North Chicago, IL, USA, Epclusa^®^ Gilead, Foster City, CA, USA), whereas others are only active against certain HCV genotypes (Harvoni^®^ Gilead, Foster City, CA, USA). Checking for coinfection with HIV and HBV is advisable. In addition, children with HCV infection may activate latent HBV infection during DAA treatment. A positive test for a HBV surface antigen or a HBV core antibody may indicate coinfection. The DAAs carry a Black Box Warning for Hepatitis B reactivation while treating the HCV, with risk of fulminant hepatitis, hepatic failure, and death [25]. Children with untreated chronic HCV infection should be monitored by physical examinations and have their serum aminotransferases measured every 6 to 12 months. More frequent monitoring is recommended in children with comorbidities such as coinfection with HIV or HBV infection.

Generally, serial HCV RNA testing in children is not recommended because HCV RNA detection does not correlate with disease severity or affect the timing of treatment decisions. If treatment is considered, measuring HCV RNA is helpful to confirm that the child still has an active HCV infection before treatment. Monitoring for hepatocellular carcinoma is recommended for children with cirrhosis [26], coinfection with HBV, and a history of leukemia [26,27] or other malignancies. In such situations, guidance suggests monitoring with liver ultrasonography and serum alpha-fetoprotein every 6 months [28]. In addition, successful treatment of the HCV in children with cirrhosis probably does not eliminate the risk of hepatocellular carcinoma, so continuing to monitor these children is recommended.

A liver biopsy is not routine because it does not affect treatment decisions. However, a liver biopsy may be appropriate for selected children with these conditions: comorbid diseases such as obesity; metabolic-associated steatotic liver disease; HIV coinfection; congenital heart disease with elevated right heart pressure; suspected advanced liver disease (signs of portal hypertension, cirrhosis), and uncertain diagnosis; and those on hepatotoxic medications or those undergoing chemotherapy. Currently there is no standard recommendation regarding when to use a FibroScan ^®^ 502, Echosens, Paris, France or transient elastography to assess liver fibrosis in children with HCV infection. However, a FibroScan or transient elastography might be another method to assess liver fibrosis before or after treatment with DAAs [29,30] and to differentiate mild cases from those with significant fibrosis in children with chronic viral hepatitis including HBV and HCV [31].

## 6. Management

Fulminant hepatitis or acute liver failure in acute HCV infection is rare in children [15,32]. A minority of patients clear the infection spontaneously, but the natural history in children is not well understood. There are no data showing treatment of acute HCV infection in children. However, adults with acute HCV infection should be treated upon initial diagnosis without awaiting spontaneous resolution, using a “test and treat” strategy and according to the simplified approach, if eligible. The duration of treatment may be shortened to 6–8 weeks in acute HCV infection [33]. For those patients who have acquired acute HCV from a liver transplant where the donor was HCV positive, they would benefit from 12 weeks of pan-genotypic DAA treatment [34].

For chronic HCV infection, the goal of treatment is eradicating HCV RNA by the attainment of a sustained virological response at 12 weeks after treatment (SVR12), which means checking HCV RNA at 12 weeks after completing treatment. If HCV RNA is negative at SVR12, chronic HCV infection is considered cured. DAAs are effective, well tolerated, available in oral form including pellet form for young children, and can be used for children as young as 3 years old. Hepatitis C treatment options with mechanisms of action and pharmacokinetics are summarized in Table 2. Trials of DAAs in children have led to approvals by the US Food and Drug Administration (FDA). We summarized the pediatric clinical trials in Table 3 and the treatment options and regimens in Table 4. For some children who have a relapse of HCV infection or who do not respond to pan-genotypic regimens, sofosbuvir/velpatasvir/voxilaprevir can be offered for patients aged ≥18 years old [35]. For rare cases of children with decompensated cirrhosis, a combination of sofosbuvir/velpatasvir with ribavirin would be recommended [36]. For children who might have poor compliance due to insurance issues, social instability, or who are not able to tolerate the granules, we would recommend deferring treatments for several years until it is appropriate because most children with HCV liver disease have a slow progression.

Ribavirin has no significant effects on HCV RNA levels as a single agent. Prolonging the course of ribavirin treatment does not add any benefit in terms of virologic clearance [37]. Therefore, ribavirin has been used for treatment of chronic HCV only in combination with IFN-alfa or other anti-HCV treatments. Pregnancy must be avoided both during and 6 months after treatment to avoid teratogenicity, specifically when considering ribavirin use [38]. Ribavirin should be avoided in childbearing age groups given its teratogenic effect.

**Table 2:** Pharmacology of Hepatitis C Medications [39].

Medication Class	Mechanism of Action	Generic Medication Name	Pharmacokinetics (Studied from Adult Data)
**Interferon-Based Therapies**	Initiate intracellular activity via modulation of cell cycle to cause cytotoxic effects to lymphocytes and virus-infected cells [40]	Interferon-Alpha	A: N/A (Intramuscular/Intravenous) D: Large volume of distribution M/E: Renal metabolism, elimination half-life approximately 2 h [40]
	Works against RNA viruses by inhibiting HCV polymerase, but mechanistically not fully understood [41]	Ribavirin	A: Bioavailability increased with high-fat meals D: Large volume of distribution M/E: Renal/hepatic metabolism (not CYP450 enzymes) [41]
**NS5A inhibitors**	Block lipoprotein complex formation and assembly [42,43,44]	Daclatasvir	A: Minimal effect by food D: 99% protein bound M/E: CYP3A/CYP3A4 metabolism, primarily fecal elimination, 53% unchanged in the feces [45]
		Elbasvir	A: Not changed by food D: 99.9% protein bound M/E: Oxidative metabolism via CYP3A, fecal elimination [46]
		Ledipasvir	A: Not changed by food D: >99.8% protein bound M/E: CYP1A2, CYP2C8, CYP2C9, CYP2C19, CYP2D6, CYP3A4; fecal elimination, 70% unchanged in the feces [43]
		Ombitasvir	A: Increased by food D: 99.9% protein bound M/E: Amide hydrolysis and oxidative metabolism, mainly fecal elimination [47]
		Pibrentasvir	A: Increased by food D: >99.9% protein bound M/E: No metabolism, fecal elimination [42]
		Velpatasvir	A: Increased by food D: >99% protein bound M/E: CYP2B6/2C8/3A4 metabolism, biliary excretion [48]
**NS5B Polymerase inhibitors**	Incorporation into RNA-dependent RNA polymerase to prevent nucleotides from being added to the RNA chain as a chain terminator [43,44]	Dasabuvir	A: Increased by food D: >99.5% protein bound M/E: CYP2C8/CYP3A4 metabolism, mainly fecal elimination [47]
		Sofosbuvir	A: Increased by food D: 61–65% protein bound M/E: Hepatic metabolism (not CYP), Glomerular filtration and active tubular secretion elimination [43,48]
**NS3/4 Protease inhibitors**	Prevent positive single-strand RNA from incorporating hepatitis C virus from replication of negative single-stranded RNA [49,50]	Asunaprevir	A: Increased by food, but no overall change D: >99% protein bound M/E: Oxidative metabolism via CYP3A, biliary-fecal elimination [51]
		Boceprevir	A: Increased by food, but no overall change D: 75% protein bound M/E: Oxidative metabolism via CYP3A4/3A5, primarily biliary-fecal elimination [52]
		Grazoprevir	A: Increased by food, but no overall change D: 99.8% protein bound M/E: Oxidative metabolism via CYP3A, fecal elimination [46]
		Glecaprevir	A: Increased by food D: 97.5% protein bound M/E: Secondary metabolism via CYP3A4, fecal elimination [42]
		Paraitaprevir	A: Increased by food, but no overall change D: 98.6% protein bound M/E: CYP3A4/3A5 metabolism, primarily fecal elimination [47]
		Simeprevir	A: Increased by food D: >99.9% protein bound M/E: CYP3A4 metabolism, biliary excretion to fecal elimination, 30% unchanged in the feces [49]
		Telaprevir	A: Increased by food D: 59–76% protein bound M/E: CYP3A4 plus hydrolysis, oxidation, and reduction reactions in the liver, majority fecal excretion [53]
		Voxilaprevir	A: Increased by food D: >99% protein bound M/E: CYP3A4 metabolism, biliary excretion [48]

Legend: A, absorption; D, distribution; M/E, metabolism/elimination; N/A, not applicable.

**Table 3:** New clinical trials of oral direct-acting antiviral agents in chronic HCV infection.

DAAs, Year	Sample Size	HCV Genotype	Duration	Results
Sofosbuvir and ribavirin, 2017 [54]	52 adolescents aged 12–17	2 or 3	12 (genotype 2) or 24 (genotype 3) weeks	- SVR12 was achieved by 98% of patients. - SVR12 rates were 100% (13/13) for patients with genotype 2 and 97% (38/39) for those with genotype 3.
Ledipasvir/sofosbuvir, 2017 [50]	100 adolescents aged 12–17	1	12 weeks	- SVR12 was achieved by 98% of patients.
Ledipasvir/sofosbuvir ± ribavirin, 2018 [55]	92 children aged 6 to <12 years	88: genotype 1 2: genotype 3 2: genotype 4	12 or 24 weeks, depending on HCV genotype and cirrhosis status.	- Overall SVR12 rate was 99%.- No on-treatment virologic failures or relapses occurred. - No patients discontinued treatment due to adverse effects, 70% of patients had AEs, most commonly headaches, pyrexia, and abdominal pain.
Ledipasvir/sofosbuvir, 2020 [56]	34 children aged 3 to 5 years	28: genotype 1a 5: genotype 1b 1: genotype 4		- One patient discontinued medication early on due to palatability. - 100% of patients who completed therapy achieved SVR12. - No patients discontinued treatment due to adverse effects, 74% of patients had AEs, most commonly vomiting and pyrexia.
Glecaprevir/pibrentasvir, 2020 [57] *	47 adolescent patients 12–17	1, 2, 3, or 4	8–16 weeks	- 100% achieved SVR12. - No on-treatment virologic failures or relapses occurred. - No patients discontinued treatment due to adverse effects, 70% of patients had AEs, most commonly nasopharyngitis, upper respiratory tract infection, and headaches.
Sofosbuvir plus ribavirin, 2020 [58]	54 children aged 3 to <12 years	2, 3	12 weeks	- 98% (53/54) achieved SVR12.
Ombitasvir, paritaprevir, ritonavir, and dasabuvir with ribavirin, 2020 [59]	26 children 3–11 years old	1	12 weeks	- The SVR12 (25–26) rate was 96%. - One child failed to achieve SVR12 due to non-adherence and treatment discontinuation.
Sofosbuvir-Velpatasvir, 2020 [60] *	216 children aged ≥ 3 years	All	12 weeks	- >90% achieved SVR12. - Most children did not achieve SVR12 due to lack of follow up or left the study after the dose of drug.
Glecaprevir/pibrentasvir, 2021 [61] *	80 children aged 3 to <12 years	1–6	8, 12, or 16 weeks	- 96% (77/80) achieved SVR12. - One participant relapsed by post-treatment week 4. - Two non-responders prematurely discontinued the study. - One patient discontinued treatment due to an adverse effect, 71% of patients had AEs, 29% of patients had an AE reasonably related to GP, most commonly vomiting, headaches, or diarrhea.
Sofosbuvir/velpatasvir/voxilaprevir, 2022 [35]	21 adolescents 12 to 17 years	1,2,3,4	8 weeks if DAA-naïve or 12 weeks for cirrhosis or prior DAA failure.	- 100% of patients (21 of 21) reached SVR12. - No patients discontinued treatment due to an adverse effect, 71% of patients had AEs, most commonly abdominal pain, headaches, and nausea. One patient had severe hypotension.
Elbasvir/grazoprevir, 2023 [62]	57 children aged 3 to <18 years	1 or 4	12 weeks	- All participants (100%) achieved SVR12 after completing treatment.

* Studies were the basis for FDA approval for treatment of chronic HCV.

**Table 4:** Treatment options and regimen for chronic HCV infection in children aged ≥ 3 years old in treatment-naïve or interferon-experienced patients without cirrhosis or with compensated cirrhosis in the United States.

DAAs	HCV Genotype	Duration (Weeks)	Doses
- Glecaprevir/pibrentasvir (MAVYRET)	Pan-genotypes	8	<20 kg: 150 mg/60 mg
			≥20–30 kg: 200 mg/80 mg
			≥30–45 kg: 250 mg/100 mg
			>45 kg or > 12 years old: 300 mg/120 mg
- Sofosbuvir/velpatasvir (Epclusa)	Pan-genotypes	12	<17 kg: 150 mg/37.5 mg
			17–30 kg: 200 mg/50 mg
			≥30 kg: 400 mg/100 mg
- Ledipasvir/sofosbuvir (Harvoni)	1, 4, 5, 6	12	<17 kg: 33.75 mg/150 mg
			17–35 kg: 45 mg/200 mg
			≥35 kg: 90 mg/400 mg

Adapted from Reference: [63].

## 7. Counseling

Children with active HCV infection should be counseled about transmission. Here is some important information for counseling children/adolescents and their families:

- Children with HCV should not be excluded from their household, school, or daycare because HCV is transmitted by blood, not from casual contact.
- It is not necessary to avoid sharing utensils, drinking glasses, towels, etc.
- Do not share toothbrushes, nail clippers, razors, or anything contaminated with blood.
- HCV can be detected at very low levels in saliva. The risk of transmission from a human bite is negligible.
- Cover any bleeding wounds with gauze, and gloves should be worn when covering the wound to prevent others from contacting blood.
- Do not donate blood.
- Avoid high-risk behaviors such as self-tattooing and self-piercing with shared needles.
- There is low risk of sexual transmission, however those with HIV coinfection or multiple sexual partners should be encouraged to use barrier precautions such as condoms.
- Always get treated for substance use disorder before/during/after HCV treatment for patients who use illicit drugs.
- Household surfaces or anything contaminated with blood from patients with HCV infection should be cleaned using diluted bleach, and gloves should be worn when cleaning a blood contaminated area.
- Women with HCV infection should be treated prior to pregnancy because this can eliminate the risk of transmission from mother to child.
- To minimize risk of disease progression, avoid alcohol consumption, obesity, and hepatotoxic medications, and receive immunizations against hepatitis A and B.

## 8. Long Term Monitoring

Gilead created a registry for adolescent and pediatric patients who received a Gilead DAA in Gilead-sponsored chronic HCV trials (NCT02510300), which started in October 2015 and were completed in January 2023, and looked at sofosbuvir, ledipasvir/sofosbuvir, sofosbuvir/velpatasvir, and sofosbuvir/velpatasvir/voxilaprevir. Their primary outcome was to determine the long-term safety of these agents. Secondary outcomes included subsequent HCV RNA testing for patients who relapsed following SVR for resistance, re-emergence, or persistence in resistance for 5 years after enrollment. They also reviewed growth and any changes in Tanner scores. Their results were presented in a poster presentation/abstract at the AASLD in Boston in 2023, with a publication on the horizon. They found that HCV treatment with sofosbuvir-based regimens resulted in durable SVR in children and had no impact on growth or sexual development up to 5 years post-treatment [64]. We have also included the different recommendations regarding HCV infection in children vs. adults in Table 5.

## 9. Future and Ongoing Studies

These are the ongoing research studies/clinical trials on clinicalTrials.gov:

- An evaluation of the adverse events and changes in disease activity in adult and adolescent participants with acute HCV infection during treatment with oral tablets of glecaprevir (GLE)/pibrentasvir (PIB) (NCT04903626) [66].
- An observational cross-sectional study on children with chronic kidney disease who are on regular hemodialysis in Egypt at a dialysis unit, which examines the prevalence of serological seroconversion of HCV and associated risk factors (NCT06104046) [67].
- A study of the safety, effectiveness, and clinical use of Maviret in adolescents with chronic HCV in Japan (NCT04214028) [68].
- An evaluation of the effectiveness of a sofosbuvir/daclatasvir combination for children aged ≥ 6 years old and adolescents with active HCV infection in Cambodia (NCT05992077) [69].
- An interventional Phase II/III, single-center, single-arm clinical trial to assess the pharmacokinetics, efficacy, safety, and tolerance of daclatasvir plus sofosbuvir in treatment-naïve, non-cirrhotic adolescents with chronic HCV genotype 4 infection in Egypt (NCT03540212) [70].
- An interventional, single-center, single-arm clinical trial to assess the pharmacokinetics, safety, efficacy, and acceptability of daclatasvir plus sofosbuvir in treatment-naïve children weighing between 14 and 35 kg with chronic HCV genotype 1–6 infection in Egypt (NCT05854511) [71].

## 10. Future Direction and Summary

In summary, the HCV is a serious infection that has high morbidity and mortality and is one of the two most common causes of liver transplantation in adults. The management of the HCV is costly in the US; however, obtaining a secure diagnosis, justification for treatment, and a pre-authorization to get treatment for a child with HCV is feasible. Outside the US, having access to updated reliable price information can allow countrys’ decision makers to negotiate better prices. Currently, multiple generic manufacturers are already lowering the price of sofosbuvir to increase access to the low-cost generic version [72].

Unlike in the adult population, treatment in the pediatric population requires different clinical and population health management optimization. Due to the heterogeneity of the HCV, country-specific or territory-specific and age-specific HCV prevalence estimates can help such regions to improve their HCV elimination strategies. However, if we can eliminate HCV infection in childhood, it will diminish and prevent disease progression and future spread. Universal prenatal screening will aid the identification of perinatally exposed newborns. The new direct-acting antiviral agents are very effective in curing HCV infection in children without serious side effects. There are limited reports on children with high-risk conditions, including cirrhosis, cancer, and HIV/HBV coinfection, which need further study in the future.

## Figures and Tables

**Table 1:** Studies of progression to advanced liver disease from HCV infection during childhood.

First Author, Year	Country	Study Design	Sample Size	Interesting Results
Birnbaum, A.H., 2000 [16]	USA	Case series	3 children	- The children presented with decompensated cirrhosis at the ages of 4, 6, and 11.
Goodman, Z.D., 2008 [17]	USA	Cross sectional	121 treatment- naïve children with liver biopsies	- 5 had bridging fibrosis, and 2 had cirrhosis. - The advanced fibrosis tended to be older.
Bortolotti, F., 2008 [18]	Italy	Retrospective and prospective observational	332 children	- 6 children with genotype 1a HCV progressed to decompensated cirrhosis with duration 2–15 years.
Mohan, P., 2013 [19]	USA	Retrospective	44 Children	- 13 children showed worsening fibrosis between first and second biopsies.

**Table 5:** The different recommendations for HCV infection in children vs. adults [65].

	Children	Adults
1. Screening	Screening for HCV infection is appropriate for infants/children who meet any of these criteria: - Evidence of hepatitis. - History of HCV infection in their mother. - International adoptees or refugees. - HIV infection. - Victims of a sexual assault. - History of multiple sexual partners. - History of illicit drug use.	- The US Preventive Services Task Force (USPSTF) subsequently recommended universal HCV screening for adults aged 18 to 79 years in March 2020. - In April 2020, the CDC recommended HCV screening at least once in all adults aged ≥18 years and for all pregnant persons during each pregnancy, except in settings where HCV prevalence is <0.1%.
2. HCV screening test	- HCV RNA is recommended for perinatally exposed infants and children aged 7–17 months. - An anti-HCV antibody test followed by a reflex NAT for HCV RNA (when anti-HCV is reactive) is recommended for perinatally exposed children aged ≥18 months.	- HCV antibody screening with reflex HCV RNA testing to establish the presence of active infection.
3. Treatment	- All children with HCV infection aged ≥3 years regardless of disease severity.	Adults with chronic HCV infection, including persons living with HIV who meet any of these criteria: Infected with any genotype. Have not previously received HCV treatment. Without cirrhosis or with compensated cirrhosis (Child-Pugh A) as determined by the following: - Liver stiffness >12.5 kPa by FibroScan. - FIB-4 > 3.25. - Noninvasive serologic tests include HCV FibroSure or enhanced liver fibrosis test. - Liver biopsy. - Liver nodularity or splenomegaly on imaging. - Platelet count < 150,000/mm^3^.
4. Monitoring	- Assess quantitative HCV RNA and hepatic function ≥ 12 weeks of therapy to confirm HCV is curable.	- Assess quantitative HCV RNA and hepatic function ≥ 12 weeks of therapy to confirm HCV is curable.
5. Long term follow up	- None unless the children have cirrhosis prior to DAA treatment, which is very rare.	- HCV treatment among HCV treatment-naive adults with compensated cirrhosis need to perform a liver ultrasound with or without alpha-fetoprotein every 6 months for hepatocellular carcinoma surveillance and evaluate for varices. - Advise abstinence from alcohol.
6. Drug interactions	- Less likely due to non-complicated past medical history.	- Monitor glucose in patients taking diabetes medications for hypoglycemia and International Normalized Ratio (INR) for patients using warfarin.
